# Synthetic lethality in KRas-driven cancer cells created by glutamine deprivation

**DOI:** 10.18632/oncoscience.253

**Published:** 2015-09-23

**Authors:** Suman Mukhopadhyay, Mahesh Saqcena, David A. Foster

**Affiliations:** Department of Biological Sciences, Hunter College of the City University of New York, New York, NY, USA; Department of Pharmacology, Weill-Cornell Medical College, New York, NY, USA

**Keywords:** KRas, glutamine, synthetic lethality, cancer metabolism, cell cycle

Oncogenic KRas mutations are present in about 30% of all human cancers and in more than 90% of pancreatic cancers. KRas-driven cancers have been largely resistant to therapeutic intervention and KRas itself has been considered undruggable. However, two recent studies have uncovered what may be an Achilles’ heel for cancer cells harboring activating KRAS mutations [1, 2]. These studies exploit a bypass of a late G1 glutamine (Gln)-dependent cell cycle checkpoint in cancer cells with KRAS mutations [3]. Upon Gln deprivation, KRas-driven cancer cells enter S-phase and arrest due to insufficient nucleotide biosynthesis [4]. The S-phase arrested cells are then vulnerable to the cytotoxic drugs capecitabine, paclitaxel, and rapamycin [1, 2]. Thus, Gln deprivation creates a “synthetic lethality” for capecitabine, paclitaxel, and rapamycin in KRas-driven cancer cells.

While Gln deprivation in a patient is not a plausible therapeutic option for treating human cancers, there are fundamental changes to Gln metabolism that occur in proliferating cancer cells that allow for interfering with Gln utilization. Gln is a “conditionally essential” amino acid that can be synthesized in mammalian cells, but during proliferation, Gln becomes an essential source of both nitrogen and carbon for nucleotide and fatty acid synthesis (Figure 1a). Gln utilization involves anaplerotic entry into the TCA cycle by sequential conversion to glutamate and then to the TCA cycle intermediate α-ketoglutarate. α-ketoglutarate needs replenishing because the citrate generated in the TCA cycle is shunted out of the mitochondria to the cytosol where acetyl-CoA is regenerated for fatty acid synthesis (Figure 1a). The conversion of glutamate to α-ketoglutarate in KRas-driven cancer cells involves a transamination reaction that involves the transfer of the α-amino group on glutamate to the α-keto group of oxaloacetate to generate aspartate and α-ketoglutarate [5] (Figure 1a). This conversion is catalyzed by the mitochondrial enzyme glutamate oxaloacetate transaminase 2 (GOT2). GOT2 is inhibited by aminooxyacetate (AOA), and importantly, AOA mimics Gln deprivation and causes S-phase arrest in KRas-driven cancer cells and G1 arrest in other cells [1, 2].

**Figure 1:**
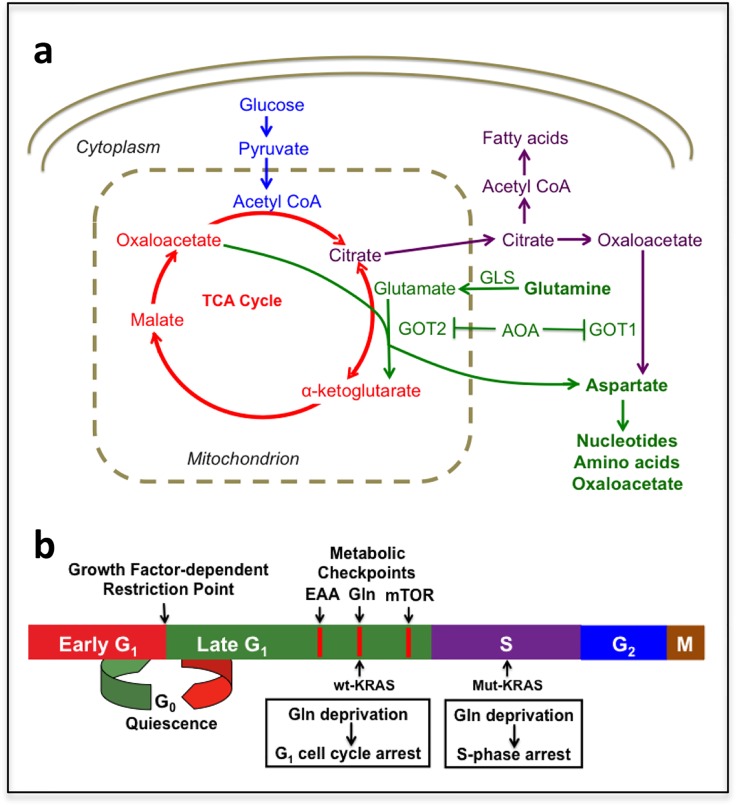
Schematic overview of anaplerotic Gln utilization and late G1 metabolic cell cycle checkpoints **A.** Gln is deaminated to glutamate by glutaminase (GLS). Glutamate is then converted to α-ketoglutarate by glutamate oxaloacetate transaminase (GOT). Aminooxyacetate (AOA) inhibits GOT and therefore suppresses generation of α-ketoglutarate from Gln-derived glutamate. The aspartate generated by GOT is critical for nucleotide and amino acid biosynthesis. **B.** The relative positions of the growth factor-dependent Restriction Point and late G_1_ metabolic checkpoints mediated by essential amino acids (EAA), Gln and mTOR are depicted.

A key point for targeting cells stuck in S-phase is the specificity for KRas-driven cancer cells – in that these cancer cells do not arrest in G1 in response to either Gln deprivation or suppression of Gln utilization. There is a distinct Gln-dependent checkpoint late in G1 that is distinguishable from the mid G1 growth factor-dependent restriction point and other late G1 metabolic checkpoints that control cell cycle progression into S-phase [3] (Figure 1b). Cancer cells harboring KRAS mutations bypass the Gln-dependent G1 checkpoint and instead are arrested in S-phase. G1 arrest in response to Gln deprivation could be restored by pharmacological suppression of the KRas downstream effectors Erk and mTOR – indicating that override of the Gln-mediated G1 checkpoint required activation of multiple KRas effector signaling pathways. There are a series of metabolic checkpoints late in G1 that prevent entry into S-phase if there are insufficient nutrients for the cell to double its mass and replicate its genome [3, 6]. A key factor for the S-phase arrest observed in Gln-deprived KRas-driven cancer cells is the production of aspartate in the transamination reaction catalyzed by GOT2. This is because aspartate is critical for nucleotide biosynthesis and the lack of aspartate generated in the GOT2 reaction leads to replicative stress due to insufficient nucleotides – which is the likely cause of the S-phase arrest. Consistent with this hypothesis, S-phase arrest could be overcome by providing both α-ketoglutarate and aspartate [1]. The α-ketoglutarate requirement is to keep the TCA cycle functioning and generating the oxaloacetate needed for aspartate production in the transamination reaction catalyzed by mitochondrial GOT2. In this regard, two recent reports have found that proliferating cells with compromised mitochondrial electron transport capability utilize cytosolic GOT1 to generate aspartate from oxaloacetate [7, 8] – reinforcing the importance of aspartate in proliferating cells.

S-phase and mitosis are the most carefully modulated phases of the cell cycle where the cell is replicating its genome and separating the chromosomes. It is during these phases where the cell is most poised to undergo apoptosis if there is something wrong. At these stages, there is no reversing the cycle and the default response to stressful conditions is generally apoptosis. Thus, the observation that KRas-driven cancer cells override a late G1 Gln-dependent checkpoint and then arrest in S-phase due to the lack of aspartate provides an exciting opportunity for therapeutic intervention in KRas-driven cancers, which have been considered undruggable. These studies also further establish the potential for exploiting metabolic changes in cancer cells that confer novel opportunities for therapeutic intervention.

## References

[1] Saqcena M (2015). Oncogene.

[2] Saqcena M (2015). Cell Cycle.

[3] Saqcena M (2013). PLoS One.

[4] Gaglio D (2009). PLoS One.

[5] Son J (2013). Nature.

[6] Foster DA (2010). Genes Cancer.

[7] Sullivan LB (2015). Cell.

[8] Birsoy K (2015). Cell.

